# Supplementary material for: Sirolimus-Embedded Silk Microneedle Wrap to Prevent Neointimal Hyperplasia in Vein Graft Model
Source: Int J Mol Sci. 2023 Feb 7;24(4):3306. doi: 10.3390/ijms24043306 (PMC9967879; doi:10.3390/ijms24043306)
Supplement: Supplementary file 1 [file ijms-24-03306-s001.zip › ijms-2157766-supplementary.pdf]

	Only Injury		Bare MN	
	2wks	4wks	2wks	4wks
NI (%)	1.95	30.50	15.80	46.00
	22.57	45.93	13.40	29.30
	22.63	39.30		28.90
	5.88	28.26		29.20
	8.93	30.51		30.90
	12.27	20.44		26.10
average	12.37	32.49	14.60	31.73
stdev	8.62	8.92	1.70	7.16

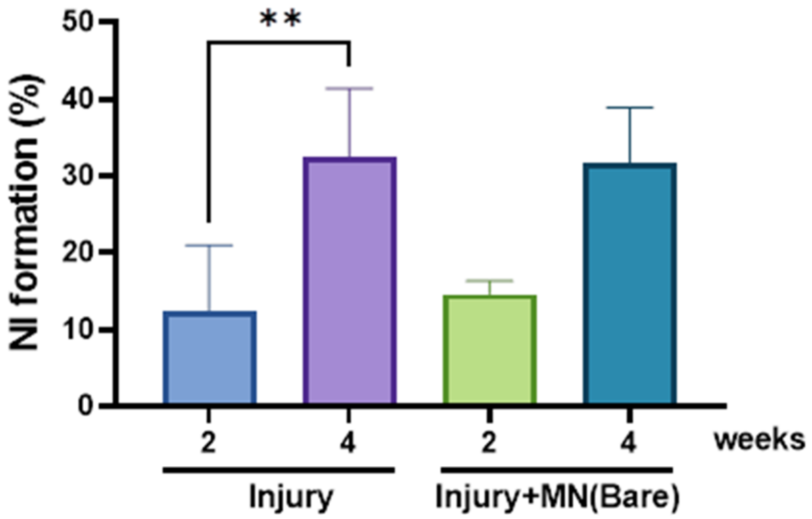

Figure S1. The percentage of neointimal hyperplasia of bare silk MN wrap without drug compared with control group without silk MN wrap in rabbit abdominal aorta injury model.
